# Disease burden of 2013-2014 seasonal influenza in adults in Korea

**DOI:** 10.1371/journal.pone.0172012

**Published:** 2017-03-09

**Authors:** Won Suk Choi, Benjamin J. Cowling, Ji Yun Noh, Joon Young Song, Seong-Heon Wie, Jin Soo Lee, Yu Bin Seo, Jacob Lee, Hye Won Jeong, Young Keun Kim, Shin-Woo Kim, Kyong-Hwa Park, Sun Hee Lee, Hee Jin Cheong, Woo Joo Kim

**Affiliations:** 1 Division of Infectious Diseases, Department of Internal Medicine, Korea University College of Medicine, Seoul, Republic of Korea; 2 WHO Collaborating Centre for Infectious Disease Epidemiology and Control, The University of Hong Kong, Pokfulam, Hong Kong Special Administrative Region, China; 3 Division of Infectious Diseases, Department of Internal Medicine, St. Vincent’s Hospital, College of Medicine, The Catholic University of Korea, Seoul, Republic of Korea; 4 Division of Infectious Diseases, Department of Internal Medicine, Inha University Hospital, Inha University School of Medicine, Incheon, Republic of Korea; 5 Division of Infectious Diseases, Department of Internal Medicine, Kangnam Sacred Heart Hospital, Hallym University College of Medicine, Seoul, Republic of Korea; 6 Division of Infectious Diseases, Department of Internal Medicine, Chungbuk University Hospital, Chungbuk National University College of Medicine, Cheongju, Republic of Korea; 7 Division of Infectious Diseases, Department of Internal Medicine, Wonju Christian Hospital, Wonju College of Medicine, Yonsei University, Wonju, Republic of Korea; 8 Department of Internal Medicine, Kyungpook National University College of Medicine, Daegu, Republic of Korea; 9 Division of Infectious Diseases, Chonnam National University Medical School, Gwangju, Republic of Korea; 10 Department of Internal Medicine, College of Medicine, Pusan National University, Busan, Republic of Korea; 11 Transgovernmental Enterprise for Pandemic Influenza in Korea, Seoul, Republic of Korea; Columbia University, UNITED STATES

## Abstract

**Background:**

This study was performed to investigate the disease burden of seasonal influenza in adults ≥20 years of age in Korea, based on surveillance data from the Hospital-based Influenza Morbidity & Mortality Surveillance (HIMM) network.

**Materials and methods:**

The HIMM network is composed of two surveillance systems: emergency room-based and inpatients-based surveillance. A total of ten university hospitals all over the country are included in the surveillance network. The adult catchment population of the HIMM network was calculated by using the data of each hospital and the database of the Health Insurance Review and Assessment Service (HIRA) of Korea. The incidence rates of laboratory-confirmed medically-attended influenza, laboratory-confirmed influenza-related admission and laboratory-confirmed influenza-related death were calculated based on the catchment population. The socioeconomic burden of influenza was estimated using the human capital approach.

**Results:**

During the 2013–2014 influenza season, the calculated adult catchment population of the HIMM network was 1,380,000. The incidence of medically-attended laboratory-confirmed influenza infection was 242.8 per 100,000 adults. The incidence of laboratory-confirmed influenza-related admission was 57.9 per 100,000 adults. The incidence of laboratory-confirmed influenza-related death was 3.1 per 100,000 adults. The total socioeconomic cost of 2013–2014 seasonal influenza in Korean adult population was estimated as 125 million USD (1 USD = 1,100 KRW).

**Conclusion:**

The disease burden of 2013–2014 seasonal influenza in Korean adult population is very high and indicates that more active prevention and control policies will be needed to decrease the burden. Additional researches will be needed to assess the burden of seasonal influenza in the Korean child population.

## Introduction

Influenza is an acute respiratory illness caused by influenza virus infection. It can cause a wide spectrum of disease from mild to severe. Influenza is associated with high morbidity and mortality especially in high-risk segments of the population. It is thought that around 5–10% of adults and 20–30% of children are infected with influenza viruses each year. As a result, annual influenza epidemics result in about 3–5 million cases of severe illness, and about 250,000–500,000 deaths in the world [1].

Epidemics of influenza occur in a different pattern depending on the region [2–4], and the burden of influenza also varies by region [5]. Korea is located in East Asian Monsoon region and has a temperate climate with four distinct seasons. The disease burden of influenza in Korea has been evaluated a few times, based on indirect analysis of National Health Insurance Claims databases or mortality time series [6–8]. Assessing disease burden of a particular disease, especially cost-based assessment, can help to understand a more realistic impact of the specific disease, and provide basic data for additional research such as cost-effectiveness analysis and related policy decisions. Previous studies on disease burden of influenza in Korea were based on health insurance data and death data. However, these data has significant limitation that the diagnostic code of influenza is inaccurate and care must be taken to interpret the results. Therefore, it is necessary to evaluate the burden of disease using actual data based on surveillance system for influenza in an institution that has experts who can diagnose influenza more sensitively and accurately. This study was performed to investigate the disease burden of 2013–2014 seasonal influenza in Korean adult population, aged 20 or more, by using surveillance data from the Hospital-based Influenza Morbidity & Mortality Surveillance (HIMM) network.

## Materials and methods

### Study sites

The HIMM network is composed of two surveillance systems: emergency room (ER)-based and inpatient-based surveillance [9]. A total of ten university hospitals all over the country are included in the surveillance network. With representativeness in mind, the hospitals of the HIMM network were selected on the basis of geographic location, population served, and emergency department capacity. Surveillance began on October 1, 2011. According to the protocol of surveillance network, ER-based surveillance was conducted using the clinical influenza-like illness criteria that is defined as sudden onset of a fever of >38’C accompanied by ≥1 respiratory symptoms (cough, sore throat, or rhinorrhea). Nasal/throat swabs were taken from compatible patients after obtaining informed consent. With the specimens, a rapid antigen test (RAT) and multiplex respiratory viral polymerase chain reaction (PCR) test were performed. Inpatient-based surveillance included monitoring for influenza-associated hospitalization, intensive care unit (ICU) admission, and mortality, and any cases of recent (within 4 weeks) laboratory-confirmed influenza were included. Owing to the surveillance system, the participating hospitals were very sensitive to the suspicion of influenza patients.

### Study participants

The study participants were adult patients 20 years of age or older, with laboratory-confirmed influenza, who visited the hospitals participating in the HIMM network during the 2013–2014 influenza season. The 2013–2014 influenza season was defined as the period from September 2013 to May 2014. Laboratory-confirmed influenza was defined as a positive result from a rapid antigen test (RAT), polymerase chain reaction (PCR) or influenza virus culture. Patients younger than 20 years of age were excluded. We excluded cases with hospital-acquired influenza, defined as laboratory-confirmed influenza where symptom onset was more than 2 days after admission.

### Calculation of adult catchment population

The adult catchment population of the HIMM network was calculated using the data of each hospital and the database of the Health Insurance Review and Assessment Service (HIRA) of Korea. It was calculated using the following formula:

Catchment population = ∑_*i*_∑_*j*_∑_*k*_*A*_*ijk*_

$$A_{ijk}=\frac{H_{ijk}}{M_{ijk}}\times P_{ijk}$$

$$\left\{\begin{array}{c} i=\text{age}\\ j=\text{gender}\\ k=area\\ {\text{A}}_{\text{ijk}}=\text{catchment population of a particular gender}/\text{age group in a particular area in}\;2012\\ {\text{H}}_{\text{ijk}}=\text{number of patients with a particular gender and age who visited the hospital A in a particular area in}\;2012\\ {\text{M}}_{\text{ijk}}=\text{number of patients with a particular gender and age who visited all medical institutions in a particular area in}\;2012\\ {\text{P}}_{\text{ijk}}=\text{number of populations with a particular gender and age in a particular area in}\;2012 \end{array}\right\}$$

The disease burden of 2013–2014 seasonal influenza was calculated using the catchment population as a denominator.

### Calculating the socioeconomic cost of influenza

We classified the cost as direct and indirect, and we used formulas from the previous research for the cost calculation [10, 11]. The baseline values used to estimate the socioeconomic cost of 2013–2014 seasonal influenza are shown in Table 1.

**Table 1:** Baseline values for calculating socioeconomic cost of 2013–2014 seasonal influenza in Korean adult population.

	Data source	Value
Direct medical cost	Database of each participating hospitals	
Average transportation cost	The report on the Korea Health Panel Survey of 2013	USD $20.12 ^a^ in 2011
Transportation cost index (ratio of 2013 *vs*. 2011)	Korean Statistical Information, Service	1.0268
Daily nursing cost	The report on the daily nursing care services institutionalization study	USD $54.55 ^a^
Correction coefficient for daily nursing cost	The report on the Korea Health Panel Survey of 2013	0.7113
Average salary of each age group and employment rate	2013 Labor employment survey by type	S1 Table

^a^ USD $1 = KRW W1,100

#### Direct cost

Direct cost, which is a cost for hospitalization or visiting outpatient clinics, was subdivided into direct medical cost and direct nonmedical cost. Direct medical cost was defined as the cost for treatment of diseases. It was calculated using the database of each hospital. Cost for purchasing medicine from pharmacy was calculated based on prescription histories and the standard price of each medicine. In terms of calculating the cost for hospitalization, if the hospital stay was extended due to problems other than influenza, the cost generated from that point to discharge was excluded from the analysis to prevent overestimation. In terms of calculating the cost for visiting clinics, if the patient received a diagnosis or treatment for problems other than influenza, the cost was excluded from the calculation.

The direct nonmedical cost consisted of transportation costs and nursing costs, and it was calculated using the following formulas:

Direct nonmedical cost=Direct nonmedical cost for visiting clinics+Direct nonmedical cost for hospitalization

$$\mathrm{Direct nonmedical cost for visiting clinics}\;=\;\sum_{m}\left(T_{m}\times \alpha \times O_{m}\right)$$

$$\left\{\begin{array}{c} \text{m}=\text{The identification number of patients who visited OPD clinics}\\ {\text{T}}_{\text{m}}=\text{Average transportation cost}\\ \alpha =\text{Crrection coeficient for transportation cost}\\ {\text{O}}_{\text{m}}=\text{Frequency of visiting outpatient clinics}\;\left(\text{days}\right) \end{array}\right\}$$

$$Direct\;nonmedical\;\cos t\;for\;hospitalization=\sum_{n}\left\{\left(T_{n}\times \alpha \right)+\left(C\times L_{n}\times \beta \right)\right\}$$

$$\left\{\begin{array}{c} \text{n}=\text{The identification number of patients who was hospitalized}\\ {\text{T}}_{\text{n}}=\text{Average transportation cost}\\ \alpha =\text{Crrection coeficient for transportation cost}\\ \text{C}=\text{Average daily nursing cost}\\ {\text{L}}_{\text{n}}=\text{Length of hospital stay}\;\left(\text{days}\right)\\ \beta =\text{Correction coefficient for nursing cost} \end{array}\right\}$$

Average round-trip transportation cost (USD $20.12 in 2011) was the value presented in ‘The report on the Korea Health Panel Survey of 2013’ [12]. The transportation costs were corrected using the transportation cost index of Korean Statistical Information, Service (KOSIS) (α = 1.0268) [13]. Because the hospitalized patients were assumed to be accompanied by a guardian, the transportation costs of those groups were calculated twice. The average daily nursing cost (USD $54.55) was the value presented in ‘The report on the daily nursing care services institutionalization study’ [14]. The nursing cost was corrected using the correction coefficient (*β* = 0.7113) because only 71.13% of hospitalized patients receive daily nursing care.

#### Indirect cost

For estimating the indirect cost, the human capital approach was used, which is based on the cost of lost workdays due to the disease. Indirect cost was calculated using the following formulas:

Indirect cost=Cost of lost workdays due to visiting clinics or hospitalization+Cost of lost income due to early death

$$\mathrm{Cost of lost work days of patients visiting clinics}\;=\;\sum_{m}\left(O_{m}\times R_{m}\times D_{m}\right)$$

$$\left\{\begin{array}{c} m=The\;identification\;number\;of\;patients\;who\;visited\;OPD\;clinics\\ {\text{O}}_{\text{m}}=\;Frequency\;of\;visiting\;outpatient\;clinics\;\left(days\right)\\ {\text{R}}_{\text{m}}=Employment\;rate\;of\;that\;age\;group\\ {\text{D}}_{\text{m}}=Average\;daily\;salary\;of\;that\;age\;group \end{array}\right\}$$

$$\text{Cost of lost work days of patinets hospitalized}=\sum_{\text{n}}\left(\gamma \times {\text{L}}_{\text{n}}\times {\text{R}}_{\text{n}}+{\text{D}}_{\text{n}}\right)$$

$$\left\{\begin{array}{c} n=The\;identification\;number\;of\;patients\;who\;was\;hospitalized\\ \gamma =Proportion\;rate\;\\ {\text{L}}_{\text{n}}=Length\;of\;hospital\;stay\;\left(\text{days}\right)\\ {\text{R}}_{\text{n}}=Employment\;rate\;of\;that\;age\;group\\ {\text{D}}_{\text{n}}=Average\;daily\;salary\;of\;that\;age\;group \end{array}\right\}$$

$$\mathrm{Cos}t\;of\;lost\;income\;due\;to\;early\;death=\sum_{s}\left[M_{s}^{t}\times \left(12-m\right)\times R_{s}^{t}+\sum_{p=1}^{65-t}\left\{\frac{M_{s}^{t+p}\times 12\times R_{s}^{t+p}}{{\left(1+\delta \right)}^{p}}\right\}\right]$$

$$\left\{\begin{array}{c} \text{s}=\text{The identification number of patients who died of influenza}\\ \text{t}=\text{Age at the time of death}\;\\ {\text{M}}_{\text{s}}^{\text{t}}=\text{Average monthly salary at the age of t years}\\ \text{m}=\text{month at the time of death}\\ {\text{R}}_{s}^{t}=\text{Employment rate at the age of t years}\\ \text{p}=\text{Time elapsed after death}\;\left(\text{years}\right)\\ \delta =\text{interest rate}\\ {\text{M}}_{\text{s}}^{\text{t}+\text{p}}=\text{Average monthly salary at the age of}\;\left(\text{t}+\text{p}\right)\;\text{years}\\ {\text{R}}_{s}^{t+p}=\text{Employment rate at the age of}\;\left(\text{t}+\text{p}\right)\;\text{years} \end{array}\right\}$$

The data of ‘2013 Survey report on labor conditions by employment type ' was used to determine the employment rate and average daily income (S1 Table) [15]. The proportion rate (γ)was presumed to be 1/3 based on the result of previous study, which presumed that treatment of three patients in clinic is equal to that of one patient in a ward [16]. The cost of lost income due to early death was calculated at 3% interest rate. For elderly over the age of 64 years, we assumed no loss of workdays for the patients themselves, as these individuals are not usually employed.

SPSS version 20.0 (SPSS for Windows, SPSS, Chicago, IL, USA) was used for statistical analysis.

### Ethical approval

The study was performed with approval of the Institutional Review Board from each of the ten university hospitals: Korea University Ansan Hospital, Korea University Guro Hospital, Catholic University St. Vincent’s Hospital, Inha University Hospital, Hallym University Kangnam Sacred Hospital, Chungbuk National University Hospital, Wonju Christian Hospital, Kyunhpook National University Hospital, Chonnam National University Hospital and Pusan National University Hospital. Written informed consent was waived because most data were collected retrospectively by reviewing medical records.

## Results

### Surveillance results of ten hospitals during 2013–2014 influenza season

During the 2013–2014 influenza season, a total of 3,341 adult patients were diagnosed with laboratory-confirmed influenza in the ten hospitals. The patients received one or more laboratory test for influenza: 3,154 (94.4%) were positive by RAT, 707 (21.2%) were positive by PCR, and 6 (0.2%) were positive by culture. Among them, 2,400 (71.8%) were positive for influenza A, 923 (27.6%) were positive for influenza B, and 16 (0.5%) were positive for both influenza A and B. Among the patients with influenza A, 196 (5.9%) were H1N1, 239 (7.2%) were H3N2, and 2,109 (63.1%) were not subtyped. The mean age of the patients was 47.5 ± 19.2 years. Among them, 1,315 (39.4%) were male, and 1,208 (36.2%) had one or more underlying diseases. Hypertension (623, 18.6%), diabetes mellitus (328, 9.8%) and chronic cardiovascular disease (240, 7.2%) were most common. Among the patients, 253 (7.6%) had influenza vaccination history at least 14 days or more from the date of symptom onset. Among the female patients, 130 (6.4%) were pregnant. Among the patients, 366 (11.0%) cases had one or more influenza-related complications and pneumonia was the most common complication (262 cases, 7.8%). Forty-three patients (1.3%) were died during hospitalization.

### Medical resource utilization pattern with influenza

The mean number (± standard deviation) of clinical visits per patient with influenza was 0.97 ± 0.8. Among the patients, 2,661 (79.6%) visited emergency department more than once, and 796 (23.8%) were hospitalized. The median duration of hospitalization was 5 days (range, 1 to 382 days). Eighty-one (2.4%) patients were admitted to an ICU and the median duration of hospitalization in ICU was 6 days (range, 1 to 34 days). Among the patients, 3,043 (91.1%) were treated with antiviral agents: oseltamivir in 2,433 (72.8%), peramivir in 599 (17.9%), and zanamivir in 26 (0.8%).

### Estimation of laboratory-confirmed influenza-related morbidity and mortality

**Fig 1:**
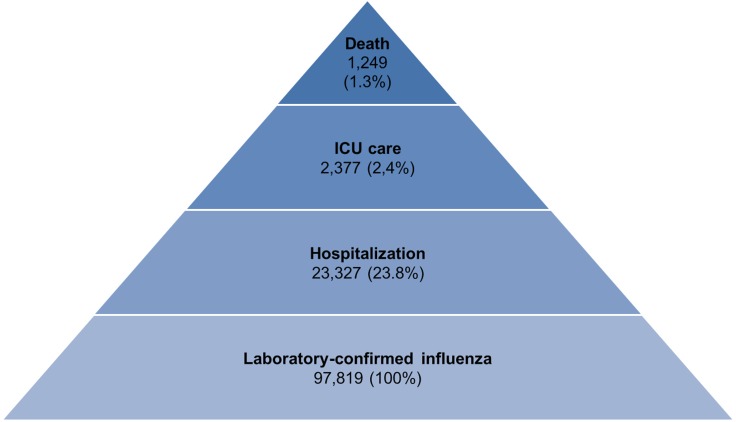
The hierarchy of the estimation of laboratory-confirmed influenza-related morbidity and mortality in Korean adult population during 2013–2014 seasonal influenza season.

In 2012, the number of adult patients using the medical facilities in Korea was 36,074,922. The number of adult population in 2012 was 40,287,814. The calculated adult catchment population of the ten hospitals was 1,380,000 (S2 Table). Using the calculated catchment population as a denominator, the estimated incidence of laboratory-confirmed influenza infection was 242.8 per 100,000 adults; the estimated incidence of laboratory-confirmed influenza-related admission was 57.9 per 100,000 adults; the estimated incidence of laboratory-confirmed influenza-related death was 3.1 per 100,000 adults (Fig 1).

### Estimation of socioeconomic cost

The average direct medical cost per one influenza patient was USD $868.7 ± 2,088.6. The average medical cost for the outpatient was USD $220.9 ± 457.7 and for the hospitalized patient was USD $3,104.3 ± 4,638.1. The direct medical cost was estimated as USD $84.9 million. Direct non-medical cost was USD $100,037,533: transportation cost, USD $3,404,847 and daily nursing cost, USD $6,632,668. Indirect cost was USD $ 299,973,196, which was a quarter of the total cost: cost of lost workdays, USD $4,782,644 and cost of lost income due to early death, USD $ 25,190,552. The total socioeconomic cost was estimated as USD $125 million (Table 2). Direct medical costs accounted for the largest portion of total costs. When the analysis was confined to the severe patients who were hospitalized, the direct medical cost was USD $66.7 million and the total socioeconomic cost was USD $104 million.

**Table 2:** The socioeconomic cost of 2013–2014 seasonal influenza in Korean adult population.

Composition of Socioeconomic Cost			Estimated Cost (USD) ^*a*^
Direct cost	Direct medical cost		84,941,875
	Direct non-medical cost	Transportation cost	3,404,847
		Daily nursing cost	6,632,686
Indirect cost	Cost of lost workdays		4,782,644
	Cost of lost income due to early death		25,190,552
Total			124,952,604

^a^ USD $1 = KRW W1,100.

## Discussion

The disease burden of seasonal influenza in Korean adult population was estimated to be very high, compared with other countries. In a study performed in the United States, the annual influenza epidemics resulted in an average of 610,660 life-years lost, 3.1 million hospitalized days, 31.4 million outpatient visits, and USD $87.1 billion of total economic burden [17]. In a study preformed in England, 779,000–1,164,000 general practice consultations, 19,000–31,200 hospital admissions, and 18,500–24,800 deaths annually were estimated to be attributable to influenza virus infections [18]. In the study performed in Italy, the estimated costs of seasonal epidemics from 1999–2008 in Italy ranged from EUR €15 to EUR €20 billion [19]. Differences in research methods, population and health care system including medical insurance system may have caused the difference of seasonal influenza disease burden.

Korea has large supply of influenza vaccine [20]. Influenza vaccination coverage is high especially in the elderly due to government reimbursement [21]. The 2013–2014 season influenza vaccine effectiveness was estimated 35–61% according to the region [22]. In this study, the effect of influenza vaccine was not considered. The actual burden of seasonal influenza may be higher than this, but it is possible that the vaccine has reduced it. However, given the high influenza vaccination rate in Korea, it is still a matter of concern that influenza cases so high burden. Considering that there has been a controversy over the influenza vaccine effect especially on A/H3N2. it is necessary to continuously evaluate whether the influenza vaccine policy in Korea is effectively influencing the relief of influenza disease burden.

This study differs from previous studies in Korea because it is based on observations from surveillance data. The previous study was performed on the basis of National Health Insurance Claims database [6, 7]. The insurance claims database can cover almost all population of Korea, but the accuracy of the influenza diagnostic code can be poor, potentially leading to underestimation of disease burden.

In the previous study performed in Korea, the burden of seasonal influenza was estimated as USD $44.7 million in 2007–2008 season and USD $42.3 million in 2008–2009 season [6]. Indirect costs accounted for about two third of total cost and the largest portion of total burden was productivity losses due to morbidity of outpatient. In this study, however, the total socioeconomic cost was estimated as USD $124.9 million, even though the analysis was confined to the adult populations. The majority of the cost was direct medical cost. The difference may be caused by low rate of laboratory-confirmation of influenza and giving the precise diagnostic codes for influenza. In particular, most of the severe patients might be diagnosed as other diseases such as sepsis, pneumonia or chronic medical diseases. It means that the national insurance data would have significant limitation to estimate the actual influenza disease burden. Another cause of the difference might be the characteristics of the HIMM surveillance system. The participating hospitals of HIMM surveillance system were university hospitals. As a result, the severity and the direct medical cost might be overestimated. This is the inevitable limitation of this study. However, the overestimation of direct medical costs was considerably controlled by excluding the cost generated by problems other than influenza. The difference in the severity caused by circulation virus strains and vaccine mismatch may have also affected the difference in disease burden.

The study on the influenza-associated excess mortality in Korea found that influenza was associated with an average of 2,900 excess deaths per year during 2003–2013 [8]. The overall all-cause excess annual mortality rate per 100,000 people was 5.97 (2.04–18.76). The study was performed using the multiple linear regression models. In this study, the mortality of laboratory-confirmed influenza was assessed as 3.1 per 100,000 adult population. Although these results may not be directly comparable, the influenza-related death in this study was estimated as almost half of the previous assessment. Besides some reasons, which is the difference in the research methods, study population, seasonal patterns of influenza epidemics and whether the 2009 pandemic influenza included, mortality in patients with undiagnosed influenza may explain the difference.

This study included only laboratory-confirmed influenza. Considering that the many patients are not confirmed by laboratory, real burden will be higher than this. In addition, the burden in child population was not included in this study. Considering that the incidence of influenza is usually higher in children than adult, it will be measured more higher if the burden of child population is included.

This study has some limitation. First, all the participating hospitals were university hospitals, and patients presenting to the study hospitals might differ somewhat from patients presenting to other hospitals. Second, the burden of seasonal influenza will vary depending on the influenza season, and the winter 2013–14 was dominated by influenza A which tends to have greater impact on adults than influenza B.

In conclusion, the disease burden of 2013–2014 seasonal influenza in Korean adult population was very high and indicates that more active prevention and control policies will be needed to decrease the burden. Further research will be needed to assess the burden of seasonal influenza in the pediatric population.

## Conclusions

The disease burden of 2013–2014 seasonal influenza in Korean adult population is very high and indicates that more active prevention and control policies will be needed to decrease the burden. Additional researches will be needed to assess the burden of seasonal influenza in the Korean child population.

## Supporting information

S1 Table Daily salary and employment rate of Korea in 2013. (DOCX)

S2 Table Adult catchment population of the participating hospitals in 2012. (DOCX)
