# Supplementary material for: Disease burden of 2013-2014 seasonal influenza in adults in Korea
Source: PLoS One. 2017 Mar 9;12(3):e0172012. doi: 10.1371/journal.pone.0172012 (PMC5344334; doi:10.1371/journal.pone.0172012)
Supplement: S1 Table — (DOCX) [file pone.0172012.s001.docx]

**S1 Table. Daily salary and employment rate of Korea in 2013.** ^a^

| Age group |  | Monthly regular &  overtime payment ($) ^b^ | Daily regular  payment ($) ^b^ | Employment rate (%) |
| --- | --- | --- | --- | --- |
| All |  | 2,080.00 | 69.33 | 59.5 |
| Age ≤ 29 years |  | 1,492.73 | 49.76 | 56.8 |
| 30 years ≤ Age ≤ 39 years |  | 2,190.91 | 73.03 | 73.2 |
| 40 years ≤ Age ≤ 49 years |  | 2,386.36 | 79.55 | 78.4 |
| 50 years ≤ Age ≤ 59 years |  | 2,166.36 | 75.55 | 73.1 |
| 60 years ≤ Age |  | 1,611.82 | 53.73 | 38.4 |

^a^ This table was created by the reconstitution of the government report; ‘2013 Survey report on labor conditions by employment type '.

^b^ USD $1 = KRW \1,100
