# Supplementary material for: Disease burden of 2013-2014 seasonal influenza in adults in Korea
Source: PLoS One. 2017 Mar 9;12(3):e0172012. doi: 10.1371/journal.pone.0172012 (PMC5344334; doi:10.1371/journal.pone.0172012)
Supplement: S2 Table — (DOCX) [file pone.0172012.s002.docx]

**S2 Table. Adult catchment population of the participating hospitals in 2012.**

| Hospital | Catchment adult population |
| --- | --- |
| A | 171,200 |
| B | 105,707 |
| C | 112,239 |
| D | 177,005 |
| E | 149,695 |
| F | 111,439 |
| G | 119,090 |
| H | 148,482 |
| I | 165,498 |
| J | 115,459 |
| Total | 1,375,814 |
